# How does fresh food prescribing fit into the social service landscape? A qualitative study in Ontario, Canada

**DOI:** 10.24095/hpcdp.44.6.03

**Published:** 2024-06

**Authors:** Laura Jane Brubacher, Matthew Little, Abby Richter, Warren Dodd

**Affiliations:** 1 School of Public Health Sciences, University of Waterloo, Waterloo, Ontario, Canada; 2 School of Public Health and Social Policy, University of Victoria, Victoria, British Columbia, Canada; 3 Guelph Community Health Centre, Guelph, Ontario, Canada; 4 The SEED, Guelph, Ontario, Canada

**Keywords:** food prescribing, social services, food insecurity, food access programs, qualitative research

## Abstract

**Introduction:**

Food prescription programs are part of the broader social prescribing movement as an approach to address food insecurity and suboptimal diet in health care settings. These programs exist amid other social services, including income-based supports and food assistance programs; however, evaluations of the interactions between these programs and pre-existing services and supports are limited. This study was embedded within a larger evaluation of the 52-week Fresh Food Prescription (FFRx) program (April 2021–October 2022); the objective of this study was to examine how program participation influenced individuals’ interactions with existing income-based supports and food assistance programs.

**Methods:**

This study was conducted in Guelph, Ontario, Canada. One-to-one (n=23) and follow-up (n=10) interviews were conducted to explore participants’ experiences with the program. Qualitative data were analyzed thematically using a constant comparative analysis.

**Results:**

Participants described their experience with FFRx in relation to existing income-based supports and food assistance programs. FFRx reportedly extended income support further to cover living expenses, allowed participants to divert income to other necessities, and reduced the sacrifices required to meet basic needs. FFRx lessened the frequency of accessing other food assistance programs. Aspects of FFRx’s design (e.g. food delivery) shaped participant preferences in favour of FFRx over other food supports.

**Conclusion:**

As food prescribing and other social prescribing programs continue to expand, there is a need to evaluate how these initiatives interact with pre-existing services and supports and shape the broader social service landscape.

**Highlights**

- This study examined how a food prescription program interacts with pre-existing services.
- Participants shared experiences with the program as related to other income-based supports and food assistance programs.
- For income-based supports: the Fresh Food Prescription (FFRx) program enabled participants to extend income further, divert it to other necessities and reduce incomerelated sacrifices.
- For food assistance programs: FFRx reduced frequency of accessing other food programs and was the preferred choice due to the program’s design (e.g. accessibility, food quality, delivery).
- As food and social prescribing initiatives expand, evaluations must consider how these programs interact with and influence the broader social service landscape.

## Introduction

Food prescription programs have emerged within the broader social prescribing movement as one approach to address food insecurity and suboptimal diet by leveraging patient–provider interactions in health care settings.^1^,^2^ Through food prescription programs, primary care providers often identify eligible patients, and then prescribe healthy foods that are subsidized or no-cost. Eligibility in food prescription programs is typically dependent on individual patients concurrently experiencing food insecurity and diet-related chronic disease.^3^ In many cases, healthy food is made available through credit or vouchers that are redeemable for various food items. Many food prescription programs offer complementary supports including access to a nutritionist or dietitian and support surrounding food literacy.^4^ Previous evaluations of food prescription programs have shown that program participation is associated with improved fruit and vegetable consumption, in addition to reductions in household food insecurity.^5^-^8^

There is broad recognition that low income is a primary driver of food insecurity, and that without addressing inadequate income among participants of food prescription programs, the long-term benefits of these programs may be limited.^9^ Despite these criticisms, food prescription programs are receiving increasing public and political support, contributing to interest and enthusiasm in initiating new programs across communities in North America.^3^ In many communities, food prescription programs represent a new food support program amid a broader social welfare landscape that includes a mix of existing social services, which includes both income-based supports and food assistance programs. Due to the eligibility criteria associated with many food prescription programs, individuals who access them may also access or be eligible for a range of other social and food assistance programs and services in their community. 

While previous evaluations of food prescribing programs have focussed on participant experiences and outcomes associated with the programs themselves,^4^ few evaluations have considered how food prescribing programs interact with other (and often pre-existing) income-based supports and food assistance programs. More specifically, and in light of the criticisms of food prescription programs, it is important to consider whether, and if so, how, participation in a food prescription program may influence relationships with and use of other income-based supports and food assistance programs.

This study was embedded within an evaluation of the 52-week Fresh Food Prescription program in Guelph, Ontario, Canada. The objective of the current study was to examine how participation in a food prescription program influenced relationships, attitudes and use of existing income-based supports and food assistance programs among participants. Overall, our aim was to highlight how participation in a new food prescription program may have implications for how participants interact with other social services. Insights from this study may be relevant to other social prescribing initiatives, as it is important to assess the growth and expansion of these initiatives within their broader social welfare landscape.

## Methods

***Ethics approval***

This study received ethics approval through the University of Waterloo (Certificate #: 44233), University of Guelph (Certificate#: 19-06-040) and University of Victoria (Certificate #: 21-0060) research ethics boards.

***Study context***

This study was conducted in Guelph, Ontario, Canada. Data from 2017 to 2018 indicate that 13.9% of households in Guelph were food-insecure, which was higher than provincial (13.3%) and national (12.7%) averages during that same period.^10^ Numerous food assistance programs exist in Guelph, including a food bank, community food pantries and nonprofit organizations that provide emergency food access (e.g. Hope House, The Salvation Army).^11^-^13^ Food-insecure households are sometimes eligible for provincial social assistance, including the Ontario Disability Support Program (ODSP) and Ontario Works (OW).^14^,^15^ Both programs provide monthly income support payments to residents of Ontario who are experiencing financial insecurity. Payment amount is determined by living situation (e.g. family size, medical needs) and includes a shelter allowance and money for basic needs such as food. 

This study was part of a larger evaluation of the 52-week Fresh Food Prescription (FFRx) program, conducted in partnership with The SEED (https://theseedguelph.ca/). The SEED is a food access program of the Guelph Community Health Centre (CHC) that is dedicated to addressing food insecurity and creating food systems change in Wellington County. Participants were referred to the FFRx program by their health care provider at the CHC, then screened for eligibility. To be eligible, participants had to be classified as food-insecure (as per a one-item food security screener derived from the Household Food Security Survey Module16) and have one or more diet-related health outcomes. 

Participants who were then enrolled in the program received a food “prescription” in the form of a voucher, which was redeemable through The SEED’s online grocery store. The voucher amount was determined by household size ($10 per person per household—to a maximum of $50—per week for 52 weeks). Vouchers could be redeemed for fresh fruits and vegetables as well as other grocery items (e.g. dairy products, pantry items) available from the online store. Food options were largely consistent from week to week, though some specialty items were added on a weekly basis. Participants also had the option to phone-in orders to The SEED customer service team, available throughout the program period (interpretation services were also available), or to order in-person at Guelph CHC. 

Rolling enrolment into the program began in April 2021, with the last participants completing the 52-week program in October 2022. A total of 62 individuals agreed to participate in the FFRx program over this time period, five of whom dropped out over the course of the program (two moved away from the area; one felt they no longer required the food support; two felt the program did not meet their needs). Over 88% of the value of the vouchers was redeemed by the remaining 57 participants. Following October 2022, the program was briefly “paused” until March 2023, when additional funding was available. During the time period of the program (2021–2022), COVID-19 pandemic restrictions, combined with rising inflation and an increasingly severe housing crisis, were the backdrop to the financial and food access challenges participants experienced. 

***Data collection***

Between July and September 2022, and as each FFRx participant was nearing the end of their participation in the program, all participants remaining in the program at endline (n=57) were invited to complete a one-to-one, semistructured interview. In total, 23 participants were successfully recruited for this study in-person or by phone. Reasons for nonparticipation in an interview included time constraints, lack of interest and the presence of complex mental health needs. For convenience, most interviews were conducted on the telephone (n=18), with the exception of a few interviews that were conducted in-person (n=5). Interviews focussed broadly on participants’ experiences with FFRx. 

Additionally, follow-up interviews were conducted from May to July 2023 with information-rich participants (e.g. those who combined FFRx with other social services and food assistance programs; n=10). These participants were identified and recruited by those who conducted the initial interviews and thus had insight as to which participants accessed multiple services or programs. Follow-up interviews were conducted at the Guelph CHC, at participants’ homes, or on the telephone, based on participant preference and convenience. These interviews focussed on how participants used FFRx in combination with other social services and food assistance programs and their perception of the food prescribing program in relation to these other programs (interview guides are available on request from the authors). 

For all interviews, participants more comfortable in a language other than English were provided with interpretation. To complement the qualitative data, select data on participants’ sociodemographic characteristics and social services use were extracted from baseline surveys that were part of broader evaluation activities.

Participants provided informed, verbal consent to participate. All interviews were audio-recorded and manually transcribed verbatim. Upon completion of interviews, participants received a $30 gift card to The SEED’s online grocery store. 

***Data analysis***

Basic descriptive statistics were calculated from survey responses to summarize interview participants’ sociodemographic data and use of social services. Qualitative data were analyzed thematically using a constant comparative analysis.^17^ Initial open coding was conducted, followed by inductive line-by-line coding. Analyses integrated both initial and follow-up data from interview transcripts. NVivo software Release 1.7.1 (QSR International, Burlington, MA, US) was used for organization and retrieval of codes and coded excerpts. Codes were expanded, merged, consolidated iteratively and developed into a parsimonious codebook that fit the data.^18^ In some instances, individual quotations have been attributed to specific respondents coded as P01, P02 … P23.

## Results

***Participant characteristics***

Participants were aged 34 to 74 years. Among those interviewed, seven participants (30.4%) were receiving ODSP (Table1). Just over a third of participants (39.3%) used both ODSP and other food assistance programs (i.e. food bank) in the past year.

**Table 1:** Descriptive characteristics of interview participants (n = 23)

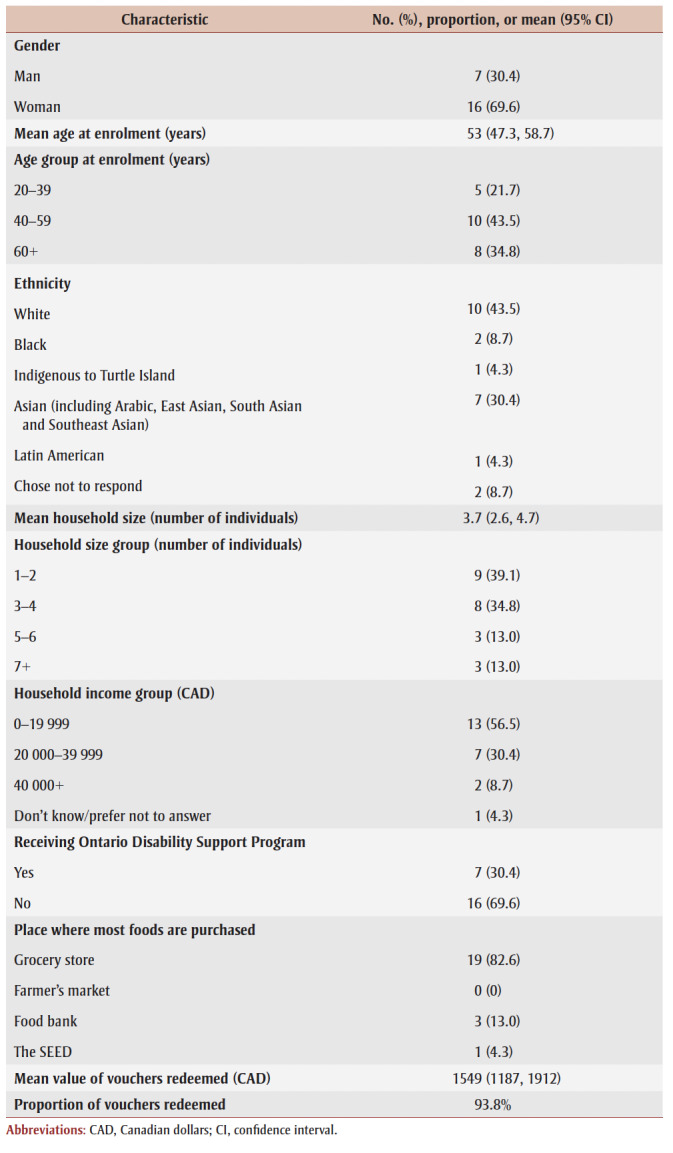

***The Fresh Food Prescription program in relation to income-based supports***

**Extending government-funded, income-based supports: “A bit of relief”**

In the context of rising food and rental costs, participants shared that government-funded, income-based supports (e.g. OW and ODSP) were often insufficient to meet their needs (P03, P08, P09, P10, P12, P16, P17). The Fresh Food Prescription program was reported, in broad terms, to extend the money participants have for expenses and provide “a bit of relief” (P17). As one participant shared, “We don’t get very much for ODSP ... FFRx gives me a little bit more money. I don’t have to pay for veggies and that, so I can support me and [my daughters]” (P03).

Another participant echoed, “[FFRx] has helped our grocery bill a lot, especially now with prices going so high” (P13). Common among participants was the need to prioritize which expenses to cover first with government-funded income support. Typically, this support was used to first pay rent and utility bills, with a small amount left for purchasing food: “[I use ODSP for] bills and rent and whatnot. So, all the things that have to be taken care of, [then] the leftovers is food” (P17). Two other participants also reported that FFRx helped them augment their income support: “[FFRx helped] to stretch a bit more ... I was able to extend everything” (P08); and “[It helped me] catch up on bills and stuff that I’ve been behind on” (P09). As one participant noted, the savings from FFRx were a helpful “supplement” when unexpected bills emerged (P16).

Furthermore, two participants noted the usefulness of FFRx in facilitating the ability to plan meals and budget expenses. As stated by one participant in relation to meal planning, “I knew, ‘I can take this $50 and spend it on this to get more meat from the grocery store, and get whatever vegetables I can grab from The SEED’” (P08). In the words of another participant: “[FFRx] helps to stay on target with your money. And you can plan your meals better knowing that you’re going to have money for that food and you can get the right nutrients at the right times” (P16). Most participants emphasized the financial challenges they experienced when FFRx was briefly paused, as funding ended, especially because they were accustomed to budgeting for expenses differently when receiving food from FFRx.

**Diverting government-funded income support elsewhere: “I’m saving that money for something else I needed”**

Similarly, participants reported that using FFRx to purchase healthy food enabled them to divert income from other sources (e.g. OW, ODSP) elsewhere. For many participants, income was diverted toward other necessities: “The money I [was] supposed to spend for groceries, it’s in my pockets. I’m saving that money for something else I needed” (P19). For example, by redeeming FFRx vouchers for fresh produce, participants could use other income to purchase meat (P04, P08, P20), or shoes or other clothing (P16, P19, P20). One participant was using the income FFRx freed up to pay for medical-related expenses, including her daughter’s transportation (via Uber) to school—as she had a serious injury and was unable to walk—as well as diabetes-related medication and supplies (metformin, needles) that were previously covered by her husband’s insurance before he was laid off (P20).

Others were able to divert income to leisure otherwise beyond their financial means, which may be important to other aspects of well-being: 

I have a little bit [of] extra money. Not a whole lot, but that little bit extra. We can spend time together, maybe go to Timmy’s [Tim Horton’s restaurant chain] or something. We couldn’t do that before because it’s pretty strapped on ODSP (P03). 

Similarly, another participant was emotional when sharing that she was able to take her son to a trampoline park with the extra savings, “and it was a good feeling that he doesn’t have to miss out on stuff ... he deserves that. You know, he hasn’t had a whole lot growing up” (P17).

**Reducing income-related compromises, trade-offs and sacrifices: “I was limiting my stuff. At least I can afford it now”**

With the financial savings created by FFRx, participants explained that they reduced some of the compromises, trade-offs and sacrifices they were accustomed to making, even while accessing other income support programs: 

I’m under the ODSP program. This program helps me to eat more healthy and is actually very helpful so I can buy the fruits I want. And everything is [getting] expensive. I was limiting my stuff. At least I can afford it now (P10). 

Prior to FFRx or during the program “pause,” one participant stated, “I bought [fresh food] for my son, but I didn’t buy for myself. There was enough for one, but not for two” (P17); another said, “I was skipping meals or skipping my fruits for the day” (P10). Moreover, a few participants’ responses suggested that expectations related to food, and experiences of compromise, changed with FFRx. For example, in reference to the pause in the FFRx program, one participant shared:

Because I didn’t have any access to the vegetables [prior to FFRx], I didn’t realize how much I missed them. When I had to introduce it into my weekly budget [when FFRx was paused], I’m thinking, “Oh my gosh, it’s either I buy this or buy this” (P03). 

It was clear from the discussion among some participants with children that expectations around food are distinct from other types of expenses, and particularly challenging to navigate with children’s needs when income is stretched:

Kids don’t understand the prices either, up [or] down. They don’t care [if] you can afford it or not. As parents, you have to provide for their needs, right? They start crying, “Give me food.” And I think they have a right to ask. But it’s hard ... without clothes, you can manage. If you [cannot] go on vacation, you can tell them like, “No, we cannot afford it. Just be patient.” But for the food, you cannot say, “Okay, stay hungry. Maybe tomorrow we can give you something” (P04). 

Overall, amid the landscape of government-funded income supports, food prescribing enabled participants to extend income support to cover basic expenses more adequately; divert income to other necessities beyond rent and bills; and reduce the trade-offs and sacrifices they were accustomed to making, given the insufficiency of government income support and a rising cost of living.

***The Fresh Food Prescription program in relation to other food assistance programs***

**Using food assistance programs in combination: “I use them in conjunction, but I don’t use them as often”**

In interviews, 13 people stated they did not access any other food assistance programs, such as the food bank, community food pantries or food assistance programs offered by nonprofit organizations, irrespective of FFRx (two were ineligible due to household income; two were unaware of other supports; two previously used supports and stopped; and seven were aware of supports but had never used them). Among interviewees, two mentioned using the food bank with the same frequency as they did prior to FFRx to access food items not available through FFRx, such as pantry products (P09, P20). Six interviewees said their frequency of accessing these other food assistance programs had changed since participating in FFRx, including two who had not used other supports at all since FFRx participation (P18, P22). 

For most participants, FFRx did not fully replace other food assistance programs, but it did shift the frequency with which users accessed them and how they prioritized these programs (P03, P08, P10, P16). The food bank was described as supplementary in relation to FFRx: “I still use them in conjunction, but I don’t use them as often ... instead of [other programs] being the main source, FFRx is my main source now” (P16). Food banks were used “more so just to put extras in the house” (P08). One participant noted, “I used to go [to the food bank] a few times a year. But since this program started, I’ve maybe used it one or two times. That’s it. For extra stuff” (P10). 

Similarly, other participants used food assistance programs in combination with FFRx to fill gaps in the program. For instance, other supports provided hygiene products or pet food not accessible through FFRx (P08) and more variety of canned food and pantry items (P03, P08, P09, P10). As one participant described, they use “the combination of everything ... different places offer different things” (P03). They went on to share what combining supports looks like in practice:

First I figure out with [FFRx]. I see what I have in my fridge. I kind of plan out what I need ... then I just sort of do it week by week. If I’m short and I can maybe get it at the food bank, then I go there. Because most of the places, you can use once a month. So I kind of stagger it so there’s always food in the house for everybody, which sometimes, without those services, I wouldn’t be able to do that (P03). 

For those participants previously accessing other food assistance programs, enrolment in FFRx shifted their interactions with those services, typically toward reduced usage. Many participants made decisions to engage first with FFRx and considered other supports as supplementary, while a few participants used them equally in combination to meet diverse food needs.

**Facilitating access to fresh fruits and vegetables: “It allows me to get fruit”**

Participants described why FFRx was largely considered their first choice within the landscape of food assistance programs, prompting them to engage differently with other services. For many participants, the FFRx program facilitated access to fresh produce they would not otherwise be able to afford amid other expenses (P03, P04, P08, P09, P10, P12, P21). As one participant shared, “It allows me to get fruit.... [Before FFRx] I’d just eat fast food or something of that nature. Processed food. So [FFRx] really was beneficial and it was much healthier” (P21). Similarly, a participant using ODSP income support explained, “You have to stretch your budget, so you’re not going to buy fresh vegetables and that. You’re going to [buy] the cheaper stuff, which is not good for you” (P03). FFRx therefore enabled participants to access healthier foods. 

In comparison to other food assistance programs, FFRx was appreciated by many participants for the higher overall quality and freshness of food they could access (P01, P15, P16, P20). For example, one participant characterized the food they receive at the food bank this way:

[The food bank provides] more than enough to keep you alive for the month, and there is a lot of frozen things ... the food bank gives you staples that have a longer shelf life and you won’t starve. But FFRx is offering you all of the fresh fruits and vegetables (P17). 

Two participants with particular health challenges (e.g. kidney issues, digestive problems) specifically noted the high salt and sugar content of boxed and canned food typically offered by other food assistance programs and expressed their appreciation for fresh produce from FFRx (P01, P20). Others noted receiving expired food (P18, P20) and produce of lesser quality (P03, P08, P09) at the food bank. 

Additionally, the FFRx program facilitated physical access to food (via home delivery). This was reportedly a significant convenience that saved participants transportation money and time (P03, P05, P08, P10, P15), especially—as one participant noted—when living in an area without nearby grocery stores (P15). Two participants specifically mentioned they do not drive a car, so transportation was a notable barrier to other food assistance programs (P10, P16). Moreover, in response to the question of how FFRx helped financially, a participant stated: 

I knew the food was there guaranteed. I didn’t have to drive around to different grocery stores shopping for deals. There was no waste of time, no wasted mental energy, no anxiety building in between—“who’s going to have a sale and who’s not?”—the food was always there ... [if] you can’t take the bus and you can’t afford a taxi, [delivery] takes a lot of stress off my head (P17). 

Accessibility vis--vis delivery had implications for mental and physical wellness. For example, participants with agoraphobia and other complex mental health challenges echoed the benefits of home delivery, especially in comparison to the triggering social environment of some food assistance locations (P09, P16). As well, participants with complex physical health challenges also noted the significant benefit of delivery (P08, P12, P20).

Finally, participants spoke to the accessibility of FFRx (e.g. no proof of income required) as distinct from other food assistance programs. Some participants described feeling ineligible for food bank support (P04, P17, P18) or being deterred by needing to show documentation of income (P10, P16). In one participant’s words, “If you’re hungry, it should never be a ‘no’ [from a food program]” (P21). 

As participants illustrated, aspects of the FFRx program design (i.e. quality of food, delivery), as well as the program’s provision of fresh produce, which was otherwise less accessible when reliant on government income support, influenced participants’ perceptions of FFRx as the first choice amid the broader food assistance landscape.

**Communicating dignity and care: “It just makes you feel that you are treated as human”**

The FFRx program was perceived as a food assistance program amid the broader landscape that implicitly communicates dignity through the structure and operations of the program. In part, the experience of dignity for participants was linked to the flexibility of the program and the way it allowed for choice (P10, P12). Other programs were noted to have limited options that “[are] prepared for your family” (P20)—where “you sort of have to take whatever they have” (P03), “it’s whatever they have on hand” (P16), and “they’re just throwing a box at you, saying ‘there you go’” (P08). As expressed by one participant in relation to other food programs and making decisions about service use, “I don’t find things that I need, so I just rather prefer not going” (P05).

The ability to choose one’s food basket with FFRx was described as particularly important by participants who were newcomers to Canada. In reference to canned food from other programs, participants shared, “In our culture, we are not using that much. So that’s why I don’t want to use [those programs] because I don’t want to waste the food I’m receiving” (P20). Similarly, referring to the choice of fresh produce from FFRx, one participant said, “I know what to cook or what not. I’m not forced to do this or that ... it’s not changing my cultural way of eating or cooking” (P04). The fact that they were allowed this kind of choice was an important factor in individuals’ decision-making regarding food assistance program use.

Food delivery also communicated dignity to participants and was emphasized as a key distinction of FFRx among the broader service landscape: “It just makes you feel that you are treated as human, because they take the extra step to deliver all that to your home, to your doorstep ... I just feel with The SEED program, there’s so much dignity and self-esteem” (P15). This was in contrast to other food assistance programs that required being present in a physical or social environment that was uncomfortable for some participants and associated with negative experiences (P08, P09, P15). 

Moreover, the fact “… that you’re actually buying [food]” (P08; i.e. with a voucher), “you’re purchasing, you don’t feel like you’re receiving for free” (P10) was another operational feature of the FFRx program that communicated dignity in relation to other food assistance programs in which an individual is only a recipient. Finally, it was noted that FFRx communicated care for individuals receiving food, particularly through the caring demeanour of program staff and the personal connection and lack of judgment participants felt from staff (P03, P16). As one participant expressed:

The [staff are] so friendly. They don’t make you feel like you’re beneath them. And I think that’s why a lot of people don’t seek out the help, because they feel that people are going to judge them, but [FFRx staff] don’t, and that’s what makes it feel okay about using the services (P03). 

Decision-making and engagement with food assistance programs was notably complex and, as illustrated, informed by participants’ experiences of dignity and care through the program’s design and staffing, as well as participants’ reflections on how they were perceived by others within a given service or support.

**Figure 1:**
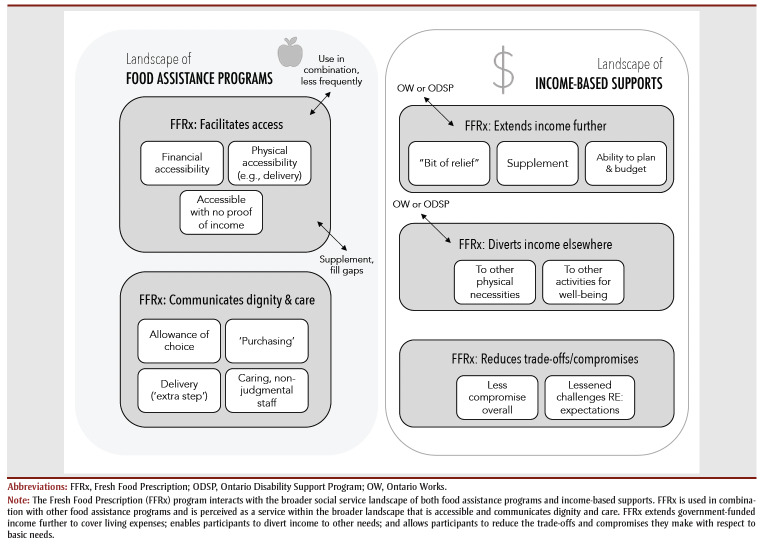
Visual synthesis of qualitative findings

Overall, participation in the FFRx program did not explicitly change attitudes towards or ability to access other food assistance programs so much as it enabled participants to change their relationship to other programs (i.e. reduce frequency of access or prioritize certain food items when accessing other programs). Their attitudes towards FFRx were expressly positive in relation to other food assistance programs, with the exception of challenges related to the program’s long-term sustainability, the shelf-life of fruits and vegetables at times, and the desire for continued expansion of the types of products offered beyond fresh produce. Aspects of the program’s design and implementation (e.g. accessibility, degree of choice of products, delivery, quality of staff interactions) made FFRx the preferred choice among all interviewed participants in relation to other food assistance programs, and consequently shifted their engagement with those services (Figure 1).

## Discussion

With growing interest in social prescribing, and food prescribing more specifically, a need exists for ongoing evaluation of these programs within the broader social service landscape. Few studies to date have examined the impact of food prescribing with this broader lens,^19^ a gap which our study aimed to address. 

Our findings add to the growing evidence that food prescribing can facilitate increased access to fresh fruits and vegetables for income-insecure individuals,^2^,^7^ allow for autonomy over food choices, and provide a sense of dignity and care that can differ from other food assistance programs.^20^-^22^ Many study participants exchanged one form of “go-to” support in favour of FFRx and reduced usage of other food assistance programs. Food prescribing was not simply another support to layer on, but an initiative that also shaped participants’ decisions regarding other services. Importantly, these findings underscore that the introduction of a food prescribing program can affect the level of interaction with other supports—that when creating a new program within a complex web of existing income-based supports and food assistance programs, individuals may make choices to engage differently with pre-existing programs. As has been discussed elsewhere,^23^,^24^ decision-making regarding social service usage is complex, informed by individuals’ experiences with and attitudes toward a given service, among many other motivations, priorities and considerations. 

These findings also have wider implications for social prescribing. New social prescribing initiatives do not emerge in isolation, but within a complex landscape of social services inclusive of income-based supports and food assistance programs. Thus, there is a need to evaluate how these new initiatives will shape individuals’ decision-making, behaviours, and interaction with other services and supports, and more fundamentally influence the broader social service landscape. 

One of the pressing critiques of food prescription programs is their inability to address the root cause of food insecurity—financial insecurity—and the assertion that resources would be more aptly directed towards income support, a living wage and broader systems-level change.^9^,^25^ We add to this discourse evidence of the benefits of these programs beyond the financial, such as the time-saving value of the FFRx program, as well as the experience of dignity and care in the provision of support. These findings relate to program design and highlight opportunities for future food prescription programs to critically assess the ways in which their programs can enhance nonmonetary benefits to participants. It may also complicate the “cash versus food” debate within food prescribing^26^ by pointing to the more nuanced benefits of these programs that factor into individuals’ engagement with and experiences of alternative services and supports.

Moreover, in relation to income security, FFRx participants reported being able to extend income support towards expenses such as rent and utility bills, divert income to other necessities such as clothing and uninsured medications, and more adequately cover basic needs rather than making difficult sacrifices and trade-offs. These findings align with that of other food prescription initiatives^7^,^23^ and also point to the ability of a food prescribing program to provide more space in a household’s discretionary budget.^27^,^28^

Thus, despite not squarely addressing the underlying determinants of food insecurity,^9^ participation in FFRx enabled a range of benefits related to income supports and financial security. Further research is needed to examine the longer-term impacts of participation in a food prescription program on financial security and in relationship to other income-based supports and services. Additionally, research is also needed to examine cost-effectiveness of food prescribing programs, recognizing the administrative costs associated with the high degree of support required (e.g. food delivery, in the case of FFRx; program staffing; and health care provider [“prescriber”] time).^26^ A recent review did not highlight delivery as a common feature of food prescribing programs.^2^ More research is required to evaluate the sustainability implications of delivery, specifically, while also considering the value of this program feature to participants, as outlined in our findings. 

***Strengths and limitations***

This study provides an in-depth examination of participant experiences with fresh food prescribing; however, it is limited to the perspectives of participants within one food prescribing program in Ontario, Canada, with access to particular income-based supports and food assistance programs. Further evaluations are needed that consider the interactions among food prescribing programs and their broader social services context. Indeed, the FFRx program was a pilot intervention within this broader social services landscape and was intended to contribute to a growing number of initiatives that aim to address food insecurity using different means. 

Additionally, there is the possibility of selection bias among our study participants. Those willing to participate may already have been more engaged with FFRx and, therefore, more likely to speak positively about the program. Moreover, the majority of study participants identified as women (n=16; 69.6%). While this may reflect to some degree the often-gendered role of women in food provision, this high proportion of women in our study limited the breadth of perspectives we may have heard from men or gender-diverse individuals. It is possible that gender could influence people’s decision-making as to what services to use and in what combination, as well as awareness of food prescribing in relation to other services and supports. Similarly, identifying as a person of a racialized group may also shape decision-making and awareness related to service use and food prescription program experience, though we did not specifically examine this within our study. These are areas for future research that would expand the current scope of available food prescribing evaluations.

## Conclusion

This study provides insight into how participation in a food prescribing program (FFRx) influenced individuals’ interactions with other income-based supports and food assistance programs. FFRx enabled participants using income-based supports to more adequately cover living expenses, afford other necessities and reduce financial sacrifices. Utilizing FFRx shifted participants’ frequency of using other food assistance programs, as food prescribing was the preferred choice due to the program’s design and participants’ experience of dignity with the support. Overall, findings from this study may be useful for other social prescribing initiatives by highlighting the value of particular program characteristics (e.g. delivery, quality of products, customizability, choice) and the need to consider the broader social services landscape, and the interaction between services, in the evaluation of new social prescribing initiatives. 

## Acknowledgements

The authors wish to express gratitude to the FFRx participants, who generously shared their perspectives and experiences with the program for this study. Funding for the FFRx research project was received from the Sprott Foundation, the McConnell Foundation, Kindred Credit Union, The City of Guelph Community Fund, MAZON Canada, a Mitacs Accelerate Grant (no. IT26188), and a Canadian Institutes of Health Research Planning and Dissemination Grant (no. 478709). Laura Jane Brubacher receives funding from a Social Sciences and Humanities Research Council of Canada Postdoctoral Fellowship Award. Matthew Little receives funding from a Michael Smith Health Research BC Scholar Award.

## Conflicts of interest

The authors declare no conflicts of interest.

Authors’ contributions and statement

LJB—conceptualization, methodology, formal analysis, writing—original draft.

ML—conceptualization, funding acquisition, writing—review and editing.

AR—conceptualization, funding acquisition, project administration, writing—review and editing.

WD—conceptualization, funding acquisition, writing—review and editing.

The content and views expressed in this article are those of the authors and do not necessarily reflect those of the Government of Canada.
